# Maximal fascicle shortening velocity measurements in human medial gastrocnemius muscle in vivo

**DOI:** 10.14814/phy2.15541

**Published:** 2023-01-03

**Authors:** Keitaro Kubo

**Affiliations:** ^1^ Department of Life Science The University of Tokyo Tokyo Japan

**Keywords:** ballistic, isokinetic, passive, plantar flexion, ultrasonography

## Abstract

This study evaluated the maximal fascicle shortening velocity under near‐no‐load conditions. In addition, we determined whether the rate of torque development during ballistic contraction was related to maximal fascicle shortening velocity. Under passive and active conditions, the medial gastrocnemius muscle fascicle shortening velocity was measured using ultrasonography at 300, 400, 500, 600, 700, 800, 900, and 1000 ° s^−1^. The maximal fascicle shortening velocity was defined as the fascicle shortening velocity under the lowest angular velocity that satisfied the following two conditions; (1) the difference in torque values between passive and active conditions was below 2.4 Nm and (2) the difference in fascicle shortening velocities between passive and active conditions was below 10 mm s^−1^. The rate of torque development was analyzed during the periods of 32, 48, 96, 152, and 200 ms after the onset of contraction during ballistic contraction. At the angular velocity (678.6 ± 147.7 ° s^−1^) that satisfied the two previously mentioned conditions, the exerted torque and the maximal fascicle shortening velocity were 1.4 ± 1.3 Nm and 251.0 ± 40.5 mm s^−1^. No significant correlations were found between the maximal fascicle shortening velocity and the rate of torque development at each time point. In conclusion, the maximal fascicle shortening velocity was quantified when the angular velocity satisfied the two conditions. Furthermore, the rate of torque development, often used as an indicator of muscle velocity, did not represent the maximal fascicle shortening velocity.

## Graphical abstract

The maximal fascicle shortening velocity of human muscles under near no‐load conditions was successfully evaluated when both the exerted torque and the difference in fascicle shortening velocities between passive and active conditions became infinitely small. In addition, the rate of torque development, often used as an indicator of muscle velocity, did not represent the maximal fascicle shortening velocity.

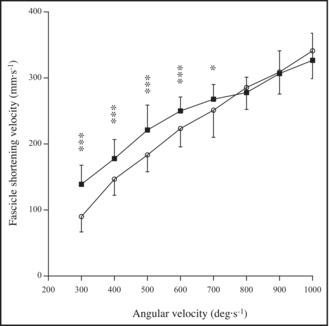

**New and Noteworthy**

Direct assessment of the maximal shortening velocity of human muscles is difficult. In the present study, the maximal fascicle shortening velocity of human muscles under near no‐load conditions was successfully evaluated when both the exerted torque and the difference in fascicle shortening velocities between passive and active conditions became infinitely small. In addition, the rate of torque development, a previously used indicator of muscle velocity, did not indicate the ability of muscles to achieve maximal fascicle shortening velocity.

## 1 INTRODUCTION

The degree to which the human skeletal muscle produces shortening velocity and force is important in sports and daily life. However, direct in vivo measurement of the maximal shortening velocity of human muscles is difficult. Previously, maximal angular velocities were estimated by extrapolation from the torque‐angular velocity relationship measured by isokinetic testing (Ferri et al., 2003; Thorstensson et al., 1976; Wickiewicz et al., 1984). This method is not optimal as the maximal angular velocity estimated is strictly different from the true maximal angular velocity at which the exerted torque is zero (Claflin & Faulkner, 1989; Desplantez & Goubel, 2002; Edman, 1988) and is further evaluated by angular velocity, rather than the shortening velocity of the muscle (Ichinose et al., 2000; Reeves & Narici, 2003). For the first time, Hauraix et al. (2015) evaluated the maximal fascicle shortening velocity of human muscles using a specifically designed dynamometer, composed of only a rotational footplate, to reduce the moment of inertia as much as possible with an ultrafast ultrasound scanner (the sampling frequency was up to 2000 Hz). In their study, a torque of 2.4 Nm (corresponding to 1.7% of maximal voluntary contraction) was exhibited, which may not be the maximal fascicle shortening velocity under no‐load conditions.

Measurement of the maximal fascicle shortening velocity under no‐load conditions may be achieved by the following two methods. Firstly, the exerted torque can be calculated by subtracting the measured torque under passive conditions (i.e., subjects were completely relaxing their muscles) from the measured torque under active conditions (i.e., subjects were contracting their muscles) to eliminate the influences of inertia and the passive element (Blanpied & Smidt, 1992; Kubo, 2014; Kubo et al., 2021; Miyamoto & Hirata, 2019; Monte & Zignoli, 2021). Therefore, we may estimate the angular velocity at which the exerted torque calculated by this method approaches 0 Nm (no‐load condition). Secondly, if the torque is exerted at a given angular velocity, the fascicle shortening velocity under active conditions should be higher than that under passive conditions. In fact, Beaumatin et al. (2017) reported that the fascicle shortening velocity during active condition was significantly higher than that during the passive condition. Therefore, the exerted torque can be considered as approaching zero (near no‐load condition) at angular velocities in which the fascicle shortening velocities of the passive and active conditions are almost equal.

Until now, the capacity to exert explosive force (corresponding to the contraction velocity of muscles) was evaluated by the rate of torque development during ballistic contraction (e.g., Aagaard et al., 2002). Previous studies demonstrated the close association between rapid muscle‐force exertion and explosive movement performance, which change with training and aging (Caserotti et al., 2008; Ema et al., 2017; Klass et al., 2008; Tillin et al., 2010). This, however, cannot be the true muscle shortening velocity under no‐load conditions, as muscle force is exerted during the measurement of torque development rate. On the contrary, some studies reported muscle fascicle behavior during explosive dynamic contractions (Hager et al., 2020; Hahn et al., 2017; Monte et al., 2020). Among them, Hager et al. (2020) reported considerably lower fascicle shortening velocity during torque development rate measurement than the maximal fascicle shortening velocity under no‐load conditions. Therefore, to adequately assess muscle maximal velocity, we must investigate the relationship between the rate of torque development and maximal fascicle shortening velocity.

The present study evaluated maximal fascicle shortening velocities under near no‐load conditions that satisfied the following two conditions: (1) the exerted torque, calculated by subtracting the measured torque under passive conditions (i.e., passive torque) from the measured torque under active conditions, was minimal, and (2) the fascicle shortening velocities under passive and active conditions were almost equal. In addition, during ballistic contraction, we determined whether the rate of torque development and fascicle shortening velocity at each time point were related to the maximal fascicle shortening velocity. The hypothesis was that maximal fascicle shortening velocities appear under angular velocity conditions that satisfy the two conditions, as described earlier, and that the rate of torque development and fascicle shortening velocity at each time point are associated with the maximal fascicle shortening velocity.

## 2 METHODS

### 2.1 Subjects

Fourteen healthy males (age: 29.4 ± 6.9 years, height: 172.7 ± 5.2 cm, weight: 69.3 ± 11.3 kg, mean ± SD) volunteered for this study. They were fully informed of the procedures as well as the study purpose with written, informed consent obtained from all subjects. This study was approved by the Ethics Committee for Human Experiments, Department of Life Science (Sports Sciences), The University of Tokyo.

### 2.2 Maximal fascicle shortening velocity under no‐load condition

Subjects lay prone on a test bench of a specially designed dynamometer (T.K.K.S‐18035, Takei Scientific Instruments Co., Ltd.), and their waist was secured by an adjustable lap belt. The foot was tightly secured by two straps to the dynamometer footplate. In addition, the toe was fixed with a strap so that it did not leave the dynamometer footplate during the measurements. The ankle joint was set at −20 deg (with the foot perpendicular to the tibia = 0 ° with positive values for plantar flexion) with the knee joint at full extension. Under passive and active conditions, the range of motion of the ankle was between −20 ° (dorsiflexed) and 25 ° (plantarflexed), and the angular velocity of the dynamometer was set at 300, 400, 500, 600, 700, 800, 900, and 1000 ° s^−1^. According to the finding of Hauraix et al. (2015), I estimated that the maximal angular velocity was around 600–700 ° s^−1^, and I decided the angular velocities measured (300–1000   s^−1^) including the individual differences among the subjects. After a standardized warm‐up, subjects performed several submaximal isokinetic contractions to get accustomed to the tests (see below). The measurements of fascicle shortening velocity at each angular velocity were performed three times per condition (passive and active). The order of tasks (300, 400, 500, 600, 700, 800, 900, and 1000 ° s^−1^) was randomized to avoid systematic effects.

**FIGURE 1:**
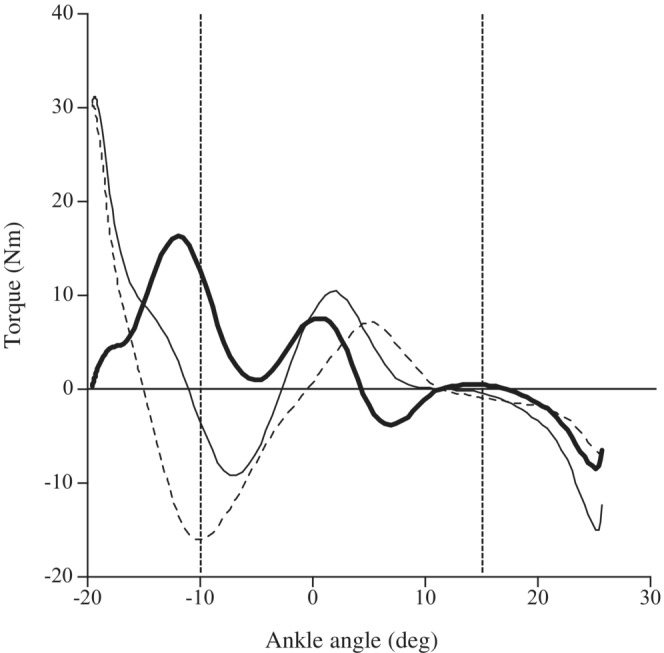
Typical examples of change in torque values during passive (dotted line) and active (thin line) conditions, and the exerted torque (thick line; the difference in torque values between passive and active conditions) at 500° s^−1^. The range within the two vertical dotted lines represents the observed range of motion (from −10° to 15°).

The analyzed range of motion was between −10 and 15°, as the mean angular velocity in this range almost matched the specified angular velocity under all angular velocity conditions. Before this measurement, three sets of trials held at −20 ° for 1 min were performed with a 1 min rest to minimize the effects of stress relaxation. Under passive conditions, within 10 s of setting the ankle angle to −20 °, the start button was pressed to start passive plantar flexion. Under active conditions, the dynamometer was programmed to begin plantar flexion when the torque value measured exceeded the value observed during the initial passive condition measurement (the passive torque within 10 s after setting to −20 ° under passive conditions). After setting the ankle angle to −20 ° and confirming a passive torque 0.3–0.5 Nm below the above‐mentioned threshold value, the subject was instructed to start plantar flexion. Accordingly, the measured torque at the start of both passive and active conditions was almost the same. The average torque of three trials during the passive conditions (caused by inertia and passive elasticity) was subtracted from the average torque of three trials during active conditions to calculate the exerted plantar flexion torque as described previously (e.g., Kubo, 2014) (Figure 1).

During the measurements, a real‐time ultrasonic apparatus (Prosound α7, Hitachi Aloka Medical) was used to continuously record longitudinal ultrasonic images of the medial gastrocnemius muscle. The scanning probe (7.5 MHz wave frequency with an 80‐mm scanning length; UST 5047‐5, Aloka) was secured, with adhesive tape and an expandable bandage, at 30% of the distance from the center of the malleolus lateralis to the articular cleft between the femur‐ and tibial condyles. Ultrasonic images were recorded at 125 Hz and synchronized to the torque and joint angle by superimposed electric signal (e.g., Kubo et al., 2020). Fascicle length was defined as the distance between the fascicle insertion into the superficial and deep aponeurosis. The fascicle length of the medial gastrocnemius muscle at −10 and 15° was analyzed, and the mean fascicle shortening velocity, over the observed range, was calculated. The fascicle length was measured five times for each image. After excluding the largest and the smallest values, the average of three measurements was used as a representative value. The maximal fascicle shortening velocity was defined as the fascicle shortening velocity under the lowest angular velocity that satisfied the following two conditions: (1) the difference in torque values between passive and active conditions (the exerted plantarflexion torque) was less than 2.4 Nm and (2) the difference in fascicle shortening velocities between passive and active conditions was less than 10 mm/s. The reasons and validity for the torque and fascicle shortening velocity thresholds are explained in the Discussion section. The coefficient of variance for the fascicle shortening velocities over three measurements was 11.4% for passive conditions and 9.3% for active conditions.

### 2.3 Fascicle shortening velocity during the measurement of the rate of torque development

Subject posture and setup were like that for the measurement of maximal fascicle shortening velocity. The ankle joint was set at 0° (anatomical position) with the knee joint at full extension. Prior to testing, the subject performed submaximal contractions to become accustomed to the test procedure. The subject was instructed to exert plantarflexion torque as fast and hard as possible. The task was repeated five times per subject with at least 2 min between trials, and the three best trials were selected for analysis. Torque onset was manually identified from the torque data by visual inspection as described previously (Tillin et al., 2010; Yamazaki et al., 2022). The rate of torque development and fascicle shortening velocity were analyzed during the periods of 32, 48, 96, 152, and 200 ms after the onset of contraction to adjust the ultrasonic images. The average value of the three measurements was then used as a representative value.

### 2.4 Statistics

Descriptive data included means ± SD. A two‐way analysis of variance (ANOVA) with repeated measures was used to detect significant effects of angular velocity and condition (passive and active) on fascicle shortening velocity. Regarding the other variables, a one‐way ANOVA with repeated measures was used to identify significant differences in the measured variables. If the *F*‐statistic of the analysis of variance was significant, differences between means were assessed using the Bonferroni post hoc test. Before the ANOVA analysis, Mauchly's sphericity test was performed to assess the homogeneity of variance and covariance. The Greenhouse–Geisser correction was used to adjust the degrees of freedom when the sphericity assumption was violated. The effect size was calculated using partial eta‐squared (*pη* ^2^) for one‐ and two‐way ANOVA. Linear regression analysis was performed on the relationship between the measured variables. The level of significance was set at *p* < 0.05.

## 3 RESULTS

**FIGURE 2:**
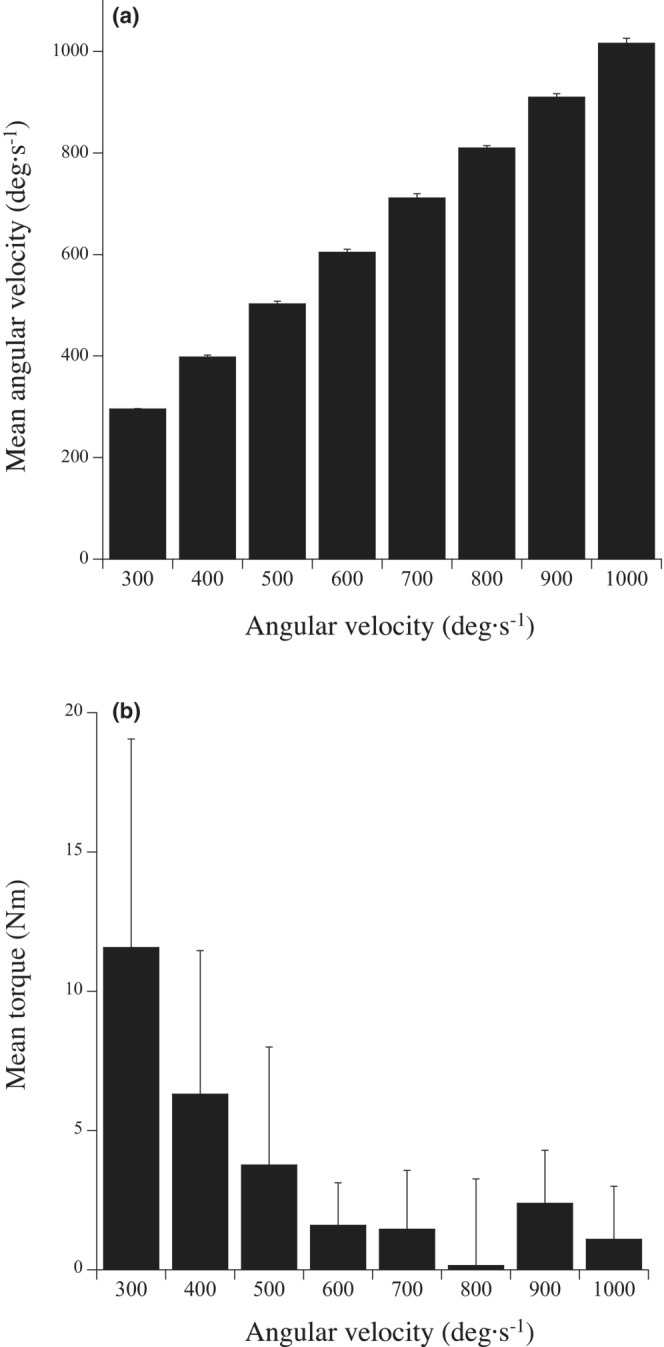
Mean angular velocity (a) and the exerted torque (b) in the observed range of motion at 300, 400, 500, 600, 700, 800, 900, and 1000° s^−1^.

Mean angular velocities and exerted plantar flexion torque (the difference in torque values between passive and active conditions) in the observed range for each angular velocity condition are shown in Figure 2. The specified angular velocity was obtained for each angular velocity condition. The mean exerted torque was less than 2.4 Nm for angular velocities of 600 ° s^−1^ or higher.

**FIGURE 3:**
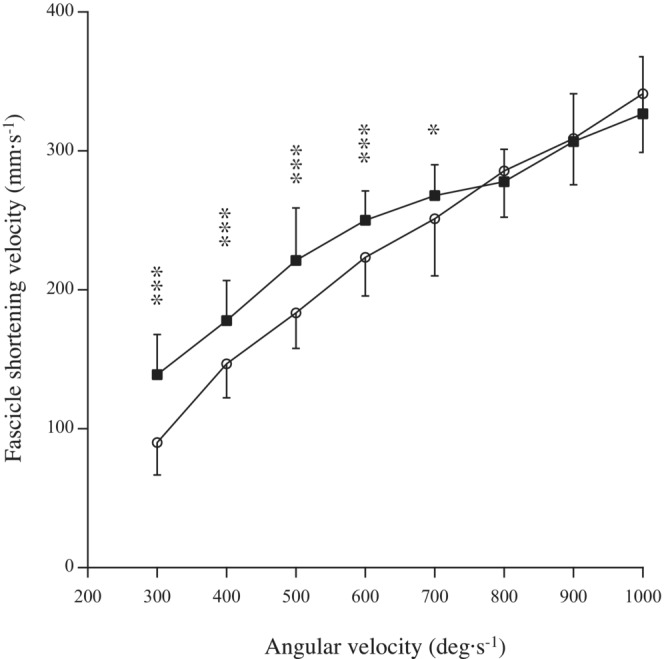
Fascicle shortening velocities under passive (open) and active (closed) conditions at 300, 400, 500, 600, 700, 800, 900, and 1000° s^−1^. Significant difference from passive conditions: *p < 0.05, ***p < 0.001.

The effects of passive or active conditions (*p* < 0.001, *pη* ^2^ = 0.796), angular velocity (*p* < 0.001, *pη* ^2^ = 0.921), and the interaction between passive or active conditions and angular velocity (*p* < 0.001, *pη* ^2^ = 0.546) were significant (Figure 3). No differences in fascicle shortening velocities for angular velocities of 800 ° s^−1^ or higher were found between passive and active conditions, whereas fascicle shortening velocities under active conditions of 700 ° s^−1^ or less were significantly higher than those under passive conditions. When the angular velocity (678.6 ± 147.7 ° s^−1^) satisfied the two conditions (the exerted torque and the difference in fascicle shortening velocities between passive and active conditions become below 2.4 Nm and 10 mm s^−1^), the exerted torque and maximal fascicle shortening velocity were 1.4 ± 1.3 Nm and 251.0 ± 40.5 mm·s^−1^ respectively.

**FIGURE 4:**
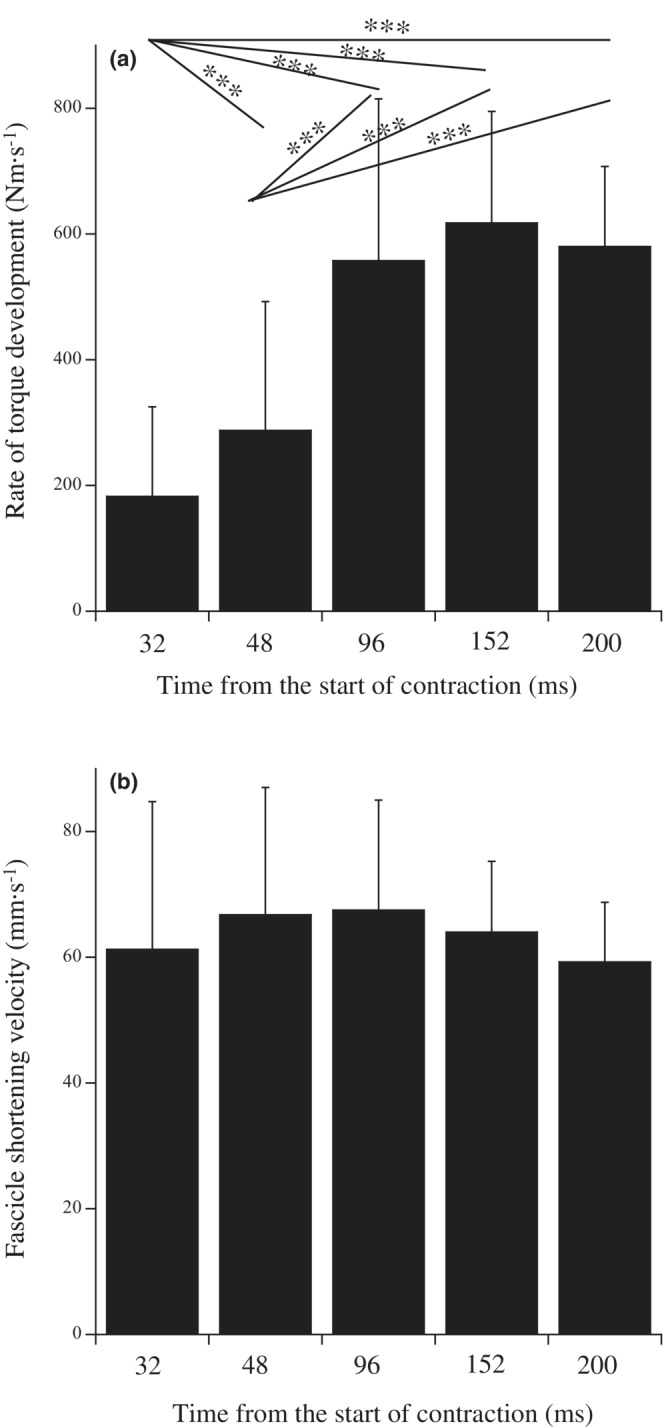
Rate of torque development (a) and fascicle shortening velocity (b) at 32, 48, 96, 152, and 200 ms from the start of contraction. Significant difference among the time points: ***p < 0.001.

**FIGURE 5:**
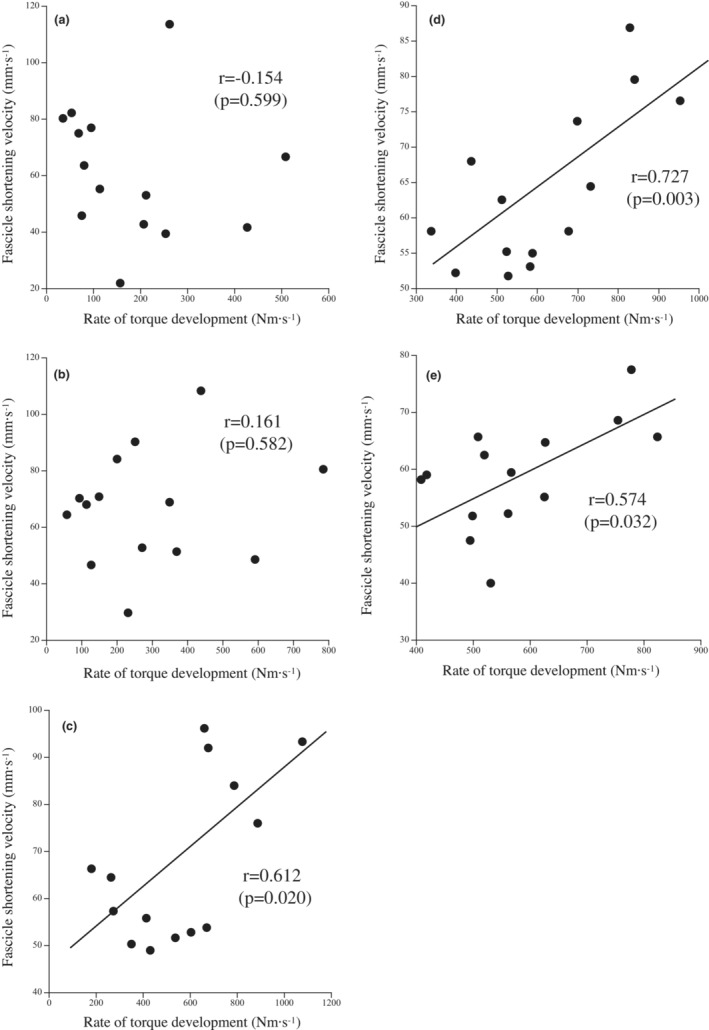
Relationships between the rate of torque development and fascicle shortening velocity at 32 (a), 48 (b), 96 (c), 152 (d), and 200 ms (e) from the start of contraction.

The rate of torque development increased with a longer observed range (*p* < 0.001, *pη* ^2^ = 0.871), but no significant difference was observed after 96 ms from the start of contraction (Figure 4a). Fascicle shortening velocity did not significantly differ within the observed ranges (*p* = 0.357, *pη* ^2^ = 0.074; Figure 4b). Significant correlations existed between torque development rate and fascicle shortening velocity at 96, 152, and 200 ms, but no significant correlations were found at 32 ms and 48 ms (Figure 5). Maximal fascicle shortening velocity was not correlated to the rate of torque development or fascicle shortening velocities at any time point (Table 1).

**TABLE 1:** Correlation coefficients between maximal fascicle shortening velocity and the measured variables

	32 sm	48 ms	96 ms	152 ms	200 ms
vs Rate of torque development	−0.044	−0.049	0.074	0.111	0.092
vs Fascicle shortening velocity	−0.291	−0.135	0.145	0.088	0.161

## 4 DISCUSSION

This study evaluated the maximal fascicle shortening velocity under the lowest angular velocity condition that satisfied the two conditions, as described earlier, of exerted torque and the passive and active fascicle shortening velocity difference. Each threshold will be discussed in the sixth paragraph of Discussion. The exerted plantarflexion torque (obtained by subtracting the passive condition torque from the active condition torque) was less than 2.4 Nm for angular velocity conditions of 600 ° s^−1^ or more. In addition, the difference between passive and active fascicle shortening velocities is less than 10 mm s^−1^ for angular velocities of 700 ° s^−1^ or more. The maximal angular velocity was, on average, 685 ° s^−1^. This value is comparable to those determined by similar methods; 648 ° s^−1^ in Hauraix et al. (2015), and 731 ° s^−1^ in Beaumatin et al. (2017). Furthermore, the average exerted torque at the maximal angular velocity was 1.4 Nm, and was lower than the value (2.4 Nm) at the no‐load condition for Hauraix et al. (2015).

As described earlier, we attempted to equalize the measured torque at the start of plantarflexion for both passive and active conditions. In other words, the exerted plantarflexion torque at the start of movement was almost 0 Nm. Previous studies did recognize the torque exerted at the start of plantarflexion. In figure 3 f of Hauraix et al. (2015), high exerted torque was observed at the start of plantarflexion under no‐load conditions, although they chose to perform contractions without pre‐activation so as not to be in a quick‐release condition. Similarly, according to table 1 of Beaumatin et al. (2017), the fascicle length under active conditions was significantly shorter than under passive conditions, which implied torque exertion at the start of plantarflexion. In previous studies, this was considered to affect the measurement of the fascicle shortening velocity (Beaumatin et al., 2017; Hauraix et al., 2015). Beaumatin et al. (2017) reported that muscle pre‐activation considerably decreased fascicle shortening velocity. This is supported as under 500 ° s^−1^, the fascicle shortening velocity when the starting movement torque was 5 Nm (179 ± 35 mm s^−1^), is significantly lower than the fascicle shortening velocity when the starting movement torque was less than 1 Nm (213 ± 32 mm s^−1^) (Kubo, unpublished data). Therefore, it is important to keep the exerted torque at the start of movement as low as possible, when measuring the maximal fascicle shortening velocity.

In the present study, the mean fascicle shortening velocity was calculated by analyzing the fascicle length of the first and last ultrasonic images within the observed range. Mean fascicle shortening velocity was, on average, 251 mm·s^−1^, comparable to reported values (284 mm·s^−1^ in ref. 23, 294 mm·s^−1^ in Hauraix et al., 2015). However, it should be noted that the maximal fascicle shortening velocity obtained here is not the instantaneous maximal value, but the average fascicle shortening velocity within a certain range of motion. Hauraix et al. (2015) quantified the instantaneous fascicle shortening velocity with an ultrafast ultrasonic device (2000 Hz), reporting an instantaneous maximum value of 340 mm s^−1^, slightly higher than the average velocity in the analysis range (290 mm s^−1^). However, considering the amount of work done by muscle fibers during exercises, the average fascicle shortening velocity within a certain analyzed range, as in this study, is considered more useful than the instantaneous maximal fascicle shortening velocity. In this study, the maximal fascicle velocity, but not the instantaneous maximal value, was quantified without the use of an ultrafast ultrasonic device.

Explosive contraction ability is an important sports performance factor (Lattier et al., 2003; Tillin et al., 2010). To date, the rate of torque development is used as a muscle velocity indicator (e.g., Aagaard et al., 2002). In addition, the rate of torque development from the onset of contraction depends on various physiological parameters, including intrinsic contractile properties, muscle fiber‐type composition, and neural activation (Andersen & Aagaard, 2006; Tillin et al., 2010). More recently, Monte and Zignoli (2021) showed that the rate of torque development was associated with both muscle and tendon stiffness. However, it is not known whether the rate of torque development reflects the fascicle shortening velocity at each time point. In the present study, the rate of torque development correlated with fascicle shortening velocities at the latter phase of muscle contraction (96, 152, and 200 ms from contraction onset), whereas those at the early phase of muscle contraction (32 and 48 ms from the onset of contraction) did not correlate. These results indicate that the rate of torque development after 100 ms from the onset of contraction represents the shortening velocity of fascicles. While the rate of torque development in the early phase of muscle contraction is influenced by other physiological variables (intrinsic contractile properties of muscle and neural drive) as suggested by Andersen and Aagaard (2006).

The rate of torque development and fascicle shortening velocity, onwards from the onset of contraction, showed no significant relationship with maximal fascicle shortening velocity (Table 1). In addition, the fascicle shortening velocities were considerably lower than the maximal fascicle shortening velocity, approximately 24% of the maximal fascicle shortening velocity. This is consistent with Hager et al. (2020), reporting fascicle shortening velocity, during the rate of torque development measurement, was 28.2% of the maximal fascicle shortening velocity. During ballistic contraction, torque is exerted unlike in a no‐load condition. Therefore, the rate of torque development only represents the fascicle shortening velocity at that time (only the latter phase) and does not show the ability of the skeletal muscle to achieve shortening velocity, i.e., the maximal fascicle shortening velocity.

To achieve no‐load conditions, the thresholds for exerted torque and fascicle shortening velocity were set by comparing these values under passive and active conditions. Regarding the exerted torque, Hauraix et al. (2015) showed that the mean torque produced over the range of motion during no‐load conditions was 2.4 Nm. Therefore, this torque value was used as the threshold value in the present study. For angular velocity conditions of 600 ° s^−1^ or more, the difference in mean torque values between passive and active conditions (the exerted torque) was less than 2.4 Nm. Regarding the fascicle shortening velocity, the differences between passive and active conditions were 15.9 mm s^−1^ for 700 ° s^−1^ and ‐7.8 mm·s^−1^ for 800 ° s^−1^ on average. In addition, the coefficient of variance for the three measurements of both conditions was around 10%. Based on the mean fascicle shortening velocity for 700 and 800 ° s^−1^ (around 270 mm s^−1^ on average) and the coefficient of variance of three measurements (10%), the fascicle shortening velocity varied by about 27 mm·s^−1^. Given this, there appears almost no difference in fascicle shortening velocity between passive and active conditions provided the difference in fascicle shortening velocity between the two conditions is less than 10 mm s^−1^.

There were several limitations to this study. First, measurements were only performed every 100 ° s^−1^ under 8 different angular velocity conditions, due to scheduling limitations and subject fatigue. To obtain more accurate maximal angular velocities and/or maximal fascicle shortening velocities, it is necessary to perform measurements with a larger number of angular velocity conditions. Second, ultrasonic images were acquired at 125 Hz, with the fascicle length measured only from the first and last ultrasonic images to calculate the mean fascicle shortening velocity during the analyzed range. Needless to say, the fascicle shortening velocity was not constant during isokinetic contractions (e.g., Ichinose et al., 2000). Unfortunately, it was impossible in this study to calculate the instantaneous maximal fascicle shortening velocity using an ultrafast ultrasonic device (2000 Hz) as in the previous studies (Beaumatin et al., 2017; Hauraix et al., 2015, 2017; Mornas et al., 2022).

In conclusion, the maximal fascicle shortening velocity of human skeletal muscles could be quantified without the use of an ultrafast ultrasonic device under the angular velocity conditions that satisfied the following; the exerted torque and the difference between passive and active fascicle shortening velocities became infinitely small. Furthermore, the rate of torque development, a muscle velocity indicator in previous studies, did not indicate maximal fascicle shortening velocity. In the future, the method presented may be used to investigate the plasticity of the ability to produce shortening velocity of human skeletal muscles to various interventions, e.g., aging, various training, and inactivity.

## CONFLICT OF INTEREST

No conflicts of interest are declared by the author.

## ETHICS STATEMENT

This study was approved by the office of the Department of Sports Sciences, The University of Tokyo, and complied with their requirements for human experimentation (approval No. 882). All subjects provided informed written consent before participation.

## References

[phy215541-bib-0001] Aagaard, P. , Simonsen, E. B. , Andersen, J. L. , Magnusson, S. P. , & Dyhre‐Poulsen, P. (2002). Increased rate of force development and neural drive of human skeletal muscle following resistance training. Journal of Applied Physiology, 93, 1318–1326. 12235031 10.1152/japplphysiol.00283.2002

[phy215541-bib-0002] Andersen, L. L. , & Aagaard, P. (2006). Influence of maximal muscle strength and intrinsic muscle contractile properties on contractile rate of force development. European Journal of Applied Physiology, 96, 46–52. 16249918 10.1007/s00421-005-0070-z

[phy215541-bib-0003] Beaumatin, N. , Hauraix, H. , Nordez, A. , Hager, R. , Rabita, G. , Guilhem, G. , & Dorel, S. (2017). Maximal shortening velocity during plantar flexion: Effects of pre‐activity and initial stretching state. Scandinavian Journal of Medicine & Science in Sports, 28, 1361–1370. 10.1111/sms.13043 29274183

[phy215541-bib-0004] Blanpied, P. , & Smidt, G. L. (1992). Human plantarflexor stiffness to multiple single‐stretch trials. Journal of Biomechanics, 25, 29–39. 1733982 10.1016/0021-9290(92)90243-t

[phy215541-bib-0005] Caserotti, P. , Aagaard, P. , Larsen, J. B. , & Puggaard, L. (2008). Explosive heavy‐resistance training in old and very old adults: Changes in rapid muscle force, strength and power. Scandinavian Journal of Medicine & Science in Sports, 18, 773–782. 18248533 10.1111/j.1600-0838.2007.00732.x

[phy215541-bib-0006] Claflin, D. R. , & Faulkner, J. A. (1989). The force‐velocity relationship at high shortening velocities in the soleus muscle of the rat. Journal of Physiology, 411, 627–637. 2614737 10.1113/jphysiol.1989.sp017595 PMC1190546

[phy215541-bib-0007] Desplantez, A. , & Goubel, F. (2002). In vivo force‐velocity relation of human muscle: A modelling from sinusoidal oscillation behaviour. Journal of Biomechanics, 35, 1565–1573. 12445609 10.1016/s0021-9290(02)00190-2

[phy215541-bib-0008] Edman, K. A. P. (1988). Double‐hyperbolic force‐velocity relation in frog muscle fibres. Journal of Physiology, 404, 301–321. 3267024 10.1113/jphysiol.1988.sp017291 PMC1190827

[phy215541-bib-0009] Ema, R. , Ohki, S. , Takayama, H. , Kobayashi, Y. , & Akagi, R. (2017). Effect of calf‐raise training on rapid force production and balance ability in elderly men. Journal of Applied Physiology, 123, 424–433. 28572499 10.1152/japplphysiol.00539.2016 PMC5583613

[phy215541-bib-0010] Ferri, A. , Scaglioni, G. , Pousson, M. , Capodaglio, P. , Van Hoecke, J. , & Narici, M. V. (2003). Strength and power changes in response to resistance training in old age. Acta Physiologica Scandinavica, 177, 69–78. 12492780 10.1046/j.1365-201X.2003.01050.x

[phy215541-bib-0011] Hager, R. , Poulard, T. , Nordez, A. , Dorel, S. , & Guilhem, G. (2020). Influence of joint angle on muscle fascicle dynamics and rate of torque development during isometric explosive contractions. Journal of Applied Physiology, 129, 569–579. 32730178 10.1152/japplphysiol.00143.2019

[phy215541-bib-0012] Hahn, D. , Bakenecker, P. , & Zinke, F. (2017). Neuromuscular performance of maximal voluntary explosive concentric contractions is influenced by angular acceleration. Scandinavian Journal of Medicine & Science in Sports, 27, 1739–1749. 28028870 10.1111/sms.12812

[phy215541-bib-0013] Hauraix, H. , Dorel, S. , Rabita, G. , Guilhem, G. , & Nordez, A. (2017). Muscle fascicle shortening behaviour of vastus lateralis during a maximal force‐velocity test. European Journal of Applied Physiology, 117, 289–299. 28044199 10.1007/s00421-016-3518-4

[phy215541-bib-0014] Hauraix, H. , Nordez, A. , Guilhem, G. , Rabita, G. , & Dorel, S. (2015). In vivo maximal fascicle‐shortening velocity during plantar flexion in humans. Journal of Applied Physiology, 119, 1262–1271. 26429868 10.1152/japplphysiol.00542.2015

[phy215541-bib-0015] Ichinose, Y. , Kawakami, Y. , Ito, M. , Kanehisa, H. , & Fukunaga, T. (2000). In vivo estimation of contraction velocity of human vastus lateralis muscle during “isokinetic” action. Journal of Applied Physiology, 88, 851–856. 10710378 10.1152/jappl.2000.88.3.851

[phy215541-bib-0016] Klass, M. , Baudry, S. , & Duchateau, J. (2008). Age‐related decline in rate of torque development is accompanied by lower maximal motor unit discharge frequency during fast contractions. Journal of Applied Physiology, 104, 739–746. 18174392 10.1152/japplphysiol.00550.2007

[phy215541-bib-0017] Kubo, K. (2014). Active muscle stiffness in the human medial gastrocnemius muscle in vivo. Journal of Applied Physiology, 117, 1020–1026. 25170073 10.1152/japplphysiol.00510.2014

[phy215541-bib-0018] Kubo, K. , Ikebukuro, T. , & Yata, H. (2020). Effect of angular velocity on active muscle stiffness in vivo. Journal of Biomechanics, 111, 110007. 32971493 10.1016/j.jbiomech.2020.110007

[phy215541-bib-0019] Kubo, K. , Ikebukuro, T. , & Yata, H. (2021). Effects of plyometric training on muscle‐tendon mechanical properties and behavior of fascicles during jumping. Physiolgical Reports, 9, e15073. 10.14814/phy2.15073 PMC8554776 34714597

[phy215541-bib-0020] Lattier, G. , Millet, G. Y. , Maffiuletti, N. A. , Babault, N. , & Lepers, R. (2003). Neuromuscular differences between endurance‐trained, power‐trained, and sedentary subjects. Journal of Strength & Conditioning Research, 17, 514–521. 12930179 10.1519/1533-4287(2003)017<0514:ndbepa>2.0.co;2

[phy215541-bib-0021] Miyamoto, N. , & Hirata, K. (2019). Muscle elasticity under active conditions in humans: A methodological comparison. Translational Sports Medicine, 2, 138–145.

[phy215541-bib-0022] Monte, A. , Baltzopoulos, V. , Maganaris, C. N. , & Zamparo, P. (2020). Gastrocnemius medialis and vastus lateralis in vivo muscle‐tendon behavior during running at increasing speeds. Scandinavian Journal of Medicine & Science in Sports, 30, 1163–1176. 32227378 10.1111/sms.13662

[phy215541-bib-0023] Monte, A. , & Zignoli, A. (2021). Muscle and tendon stiffness and belly gearing positively correlate with rate of torque development during explosive fixed end contractions. Journal of Biomechanics, 114, 110110. 33302182 10.1016/j.jbiomech.2020.110110

[phy215541-bib-0024] Mornas, A. , Racinais, S. , Brocherie, F. , Alhammoud, M. , Hager, R. , Desmedt, Y. , & Guilhem, G. (2022). Faster early rate of force development in warmer muscle: An in vivo explanation of fascicle dynamics and muscle‐tendon mechanical properties. American Journal of Physiology Regulatory, Integrative and Comparative Physiology, 323, R123–R132. 35579335 10.1152/ajpregu.00280.2021

[phy215541-bib-0025] Reeves, N. D. , & Narici, M. V. (2003). Behavior of human muscle fascicles during shortening and lengthening contractions in vivo. Journal of Applied Physiology, 95, 1090–1096. 12740314 10.1152/japplphysiol.01046.2002

[phy215541-bib-0026] Thorstensson, A. , Grimby, G. , & Karlsson, J. (1976). Force‐velocity relations and fiber composition in human knee extensor muscles. Journal of Applied Physiology, 40, 12–16. 1248977 10.1152/jappl.1976.40.1.12

[phy215541-bib-0027] Tillin, N. A. , Jimenez‐Reyes, P. , Pain, M. T. G. , & Folland, J. P. (2010). Neuromuscular performance of explosive power athletes versus untrained individuals. Medicine & Science in Sports & Exercise, 42, 781–790. 19952835 10.1249/MSS.0b013e3181be9c7e

[phy215541-bib-0028] Wickiewicz, T. L. , Roy, R. R. , Powell, P. L. , Perrine, J. J. , & Edgerton, V. R. (1984). Muscle architecture and force‐velocity relationships in humans. Journal of Applied Physiology, 57, 435–443. 6469814 10.1152/jappl.1984.57.2.435

[phy215541-bib-0029] Yamazaki, K. , Inoue, K. , & Miyamoto, N. (2022). Passive and active muscle elasticity of medial gastrocnemius is related to performance in sprinters. European Journal of Applied Physiology, 122, 447–457. 34797438 10.1007/s00421-021-04848-5

